# Genetic Determinants of the Familial Hypercholesterolaemia Phenotype

**DOI:** 10.1111/ahg.12594

**Published:** 2025-04-02

**Authors:** Steve Eric Humphries, Marta Futema

**Affiliations:** ^1^ Institute of Cardiovascular Science, Faculty of Population Health University College London London UK; ^2^ Cardiovascular and Genomics Research Institute, School of Health & Medical Sciences, City St George's University of London London UK

## Abstract

Individuals with familial hypercholesterolaemia (FH) have severely elevated plasma concentrations of low‐density lipoprotein cholesterol (LDL‐C) from birth and as a consequence have an elevated morbidity and mortality due to the development of coronary heart disease (CHD). Monogenic FH can be caused by carrying a single copy of a pathogenic variant in any of four genes (*LDLR/APOB/PCSK9/APOE)*, which are all involved in the clearance of LDL‐C from the blood by the liver. FH is one of the most common inherited disorders, with an estimated prevalence of carriers of around 1/280 individuals in most populations and ancestry groups. However, such variants can be found usually only in 20%–30% of clinically FH subjects, and in the majority of the no‐variant individuals, the phenotype is most likely explained by the inheritance of a greater‐than‐average number of common variants of small effect, with such individuals better given the diagnosis of ‘polygenic hypercholesterolaemia’. Also, in a proportion of no‐variant subjects who meet the clinical criteria, the most likely explanation is due to overproduction of Lp(a) which is an LDL‐C particle with a bound copy of the ‘little‐a’ protein. Here, we review the research that has elucidated the genetic architecture of the FH phenotype and discuss recent studies and future prospects of finding additional genes where variants can cause FH.

## 1 Introduction

One of the first descriptions of familial hypercholesterolaemia (FH; OMIM 143890) was made some 85 years ago by the Norwegian physician, Carl Müller. He described hereditary heart disease due to xanthomatosis and hypercholesterolaemia to be fairly common. It was demonstrated to be a dominant trait in families (Müller 1939).

FH is characterised by having significantly elevated total‐ and low‐density lipoprotein (LDL)‐cholesterol (LDL‐C) and premature coronary heart disease (CHD; Austin et al. 2004). The UK Simon Broome Register of FH patients has helped develop criteria that are used in the clinical diagnosis of FH. These include LDL‐C over 4.9 mmol/L in an adult and over 4.0 mmol/L in a child, plus the presence of a family history of elevated cholesterol and or a family history of premature CHD. A diagnosis of ‘Definite FH’ is given if the patient also has stigmata of elevated cholesterol of tendon xanthomas, while a diagnosis of ‘Possible FH’ is made if the patient has only high levels of cholesterol and a family history of hypercholesterolemia or premature CHD (Marks et al. 2003). A diagnostic algorithm based on the Dutch Lipid Clinic Network (DLCN) is also widely used (Marks et al. 2003). Diagnosis is based on assigning points to the different clinical FH criteria, with increasing concentrations of LDL‐C, giving up to 8 points for the highest. For individuals with a total point score of > 8, a diagnosis of Definite FH is given, with those between > 5 and 8 having a diagnosis of Probable FH, 3–5 a diagnosis of Possible FH, while those scoring below 3 do not have clinical FH. Both of these diagnostic criteria emphasise the importance of ruling out secondary causes of very high LDL‐C (due to environmental or metabolic issues such as thyroid deficiency) to improve diagnostic specificity.

As a group, individuals with a clinical diagnosis of Definite, Possible or Probable FH experience lifelong elevated LDL‐C, which if untreated, leads to an increased risk of CHD and premature death (Austin et al. 2004; Marks et al. 2003). If untreated, men with FH have a 50% risk of fatal or non‐fatal CHD by age 50 years, and women have a 30% risk by age 60 years (‘Risk of Fatal Coronary Heart Disease in Familial Hypercholesterolaemia. Scientific Steering Committee on Behalf of the Simon Broome Register Group 1991). While lifestyle and dietary changes are recommended for those with FH (Nordestgaard et al. 2013), almost all subjects with a clinical diagnosis of FH require lipid‐lowering therapy (LLT) such as a statin, to reduce LDL‐C levels to (or below) that seen in non‐FH subjects. Studies of individuals in the UK Simon Broome FH Register compared rates of CHD morbidity and mortality before and after the availability of statins and found that LLT resulted in a significant reduction in CHD mortality (Humphries et al. 2018) and morbidity (Iyen et al. 2020). Though for reasons that are still unclear, the reductions were greater in men than in women. In this current review, we focus on the genetic causes of FH. The review by Mach et al. deals with the wide range of powerful and safe pharmacological treatment options currently available for individuals FH (Mach et al. 2020).

With the use of molecular genetics techniques in individuals and families with clinical FH, it is now possible to identify in many patients the specific causative variant they carry and to give a genetic diagnosis of FH (Futema et al. 2021). Such confirmatory genetic testing is recommended by all recent guidelines (e.g., Nordestgaard et al. 2013; Mach et al. 2020), as the information can be used to assess future risk of CHD, to tailor LLT and to test at‐risk relatives to identify additional FH carriers. In the early 1980s, the Nobel Prize‐winning cellular and molecular work of Mike Brown and Joe Goldstein (Brown and Goldstein 1996) led to the identification of the LDL receptor (*LDLR*) gene as being the first gene where mutations cause the FH phenotype. We now know that autosomal dominant monogenic FH can also be caused by pathogenic variant in any of three additional genes (*APOB/PCSK9/APOE*), all of which encode proteins that have a clear role in the removal of LDL‐C from the blood by the liver. In FH mutation carriers, blood cholesterol level is on average raised two‐fold above the normal level, but there is considerable heterogeneity of effect size depending on the specific variant underlying the diagnosis. In this review, we have not described the extensive literature on genotype‐phenotype relationships, but in general, individuals carrying a complete loss‐of‐function variant have a more severe phenotype (i.e., higher LDL‐C concentration and earlier development of CHD), compared to those where the variant results in a protein with retained partial function (Futema et al. 2021).

In rare cases, families have been identified where a recessive pattern of inheritance of hypercholesterolaemia is seen (Cohen et al. 2003). In the recessive hypercholesterolaemia cases, pathogenic variants in genes that are involved in LDL‐receptor recycling (*LDLRAP1*), or other aspects of intestinal cholesterol absorption, where mutations in two genes (*ABCG5/ABCG8*) cause an elevation of plant sterols in the blood and the disorder sitosterolaemia, or hepatic lipid metabolism (*LALD*), have been identified and will not be discussed here.

Strictly speaking, FH should be regarded as a *co‐dominant* disorder, with those carrying one pathogenic variant having the diagnosis of heterozygous FH (HeFH), while those carrying two pathogenic variants have homozygous FH (HoFH). While HeFH affects 1 in 250 to 1 in 300 of the general population (Akioyamen et al. 2017), as would be expected for a Mendelian disorder, HoFH occurs in roughly three individuals per million (in randomly mating outbreeding populations; Sjouke et al. 2015). HoFH, which is a much more severe disorder (Cuchel et al. 2023), will not be covered in this review.

Currently, genetic testing in diagnostic laboratories only finds an FH‐causing variant in a minority of cases of clinical FH patients sent from lipid clinics. In the United Kingdom, between 2022 and 2023, 23,855 index cases were tested with 5126 found to carry an FH‐causing variant (a detection rate of 21.6%; Humphries et al. 2023). In general, the detection rate is higher in patients with a clinical diagnosis of Definite FH, compared to those with Possible or Probable FH, and this overall figure is a reflection of the relative proportions of these clinical FH patients in the referred cohort. While a proportion of the remainder may have a monogenic cause in a yet‐to‐be‐identified gene, recent work has demonstrated that the genetic architecture of clinical FH has a significant polygenic component. Using a genetic risk score (GRS) consisting of multiple common variants each of which is associated with a modest effect on raising LDL‐C, this polygenic component has been revealed. In addition, several different approaches have now demonstrated conclusively that variation at the *LPA* locus, which encodes the apolipoprotein (a) and leads to high plasma concentrations of the lipoprotein Lp(a), can also mimic the clinical FH phenotype. In this review, we examine these components in turn, as well as describe further potential FH‐causing genes and future genetic studies.

## 2 Genes Where Pathogenic Variants Cause Monogenic FH

### 2.1 LDLR

**FIGURE 1:**
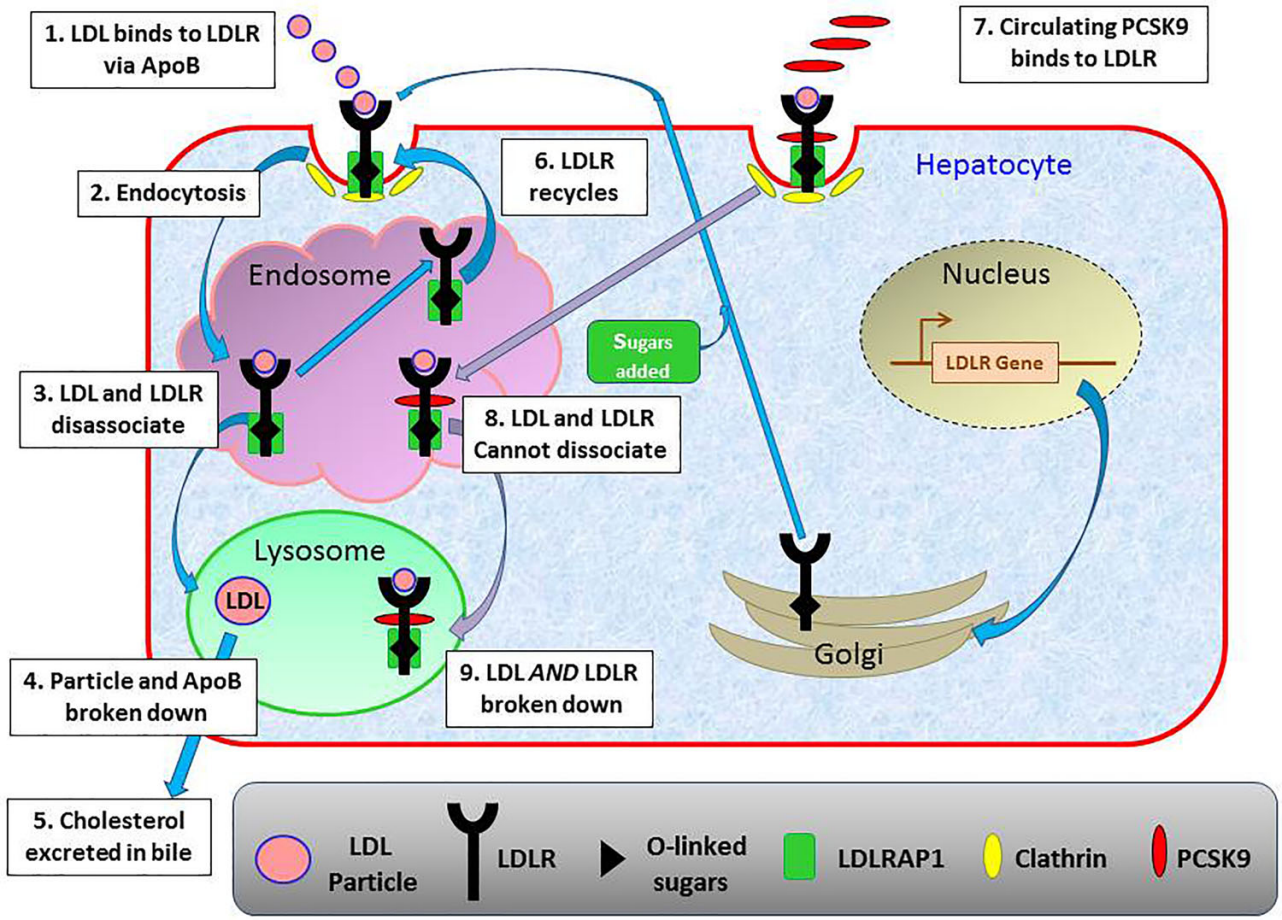
Cartoon showing the process and proteins involved in the removal of LDL‐C by the liver. (1) Lipoproteins containing either apoE or apoB (e.g., VLDL remnants and LDL) bind to the LDL receptor on the surface of liver cells. (2) The receptor‐lipoprotein complex is internalised by receptor‐mediated endocytosis and moves to the endosome compartment. (3) Here, there is a change in pH that causes the binding region of the receptor to change conformation and release the particle. (4) The particle enters the lysosome compartment where the proteins are degraded, and (5) the lipid component metabolised to bile, which is secreted into the intestine to emulsify dietary fats. (6) The LDL‐receptor recycles to the cytoplasmic membrane to repeat the process (usually up to 150 times). (7) The PCSK9 protein is secreted from the liver into the circulation and subsequently binds to the LDL‐receptor‐lipoprotein complex. (8) After internalisation, the bound PCSK9 prevents the receptor dissociating from the lipoprotein; and (9) all three components are broken down in the endosomal compartment.

Since FH is a disorder of LDL‐C metabolism, it is important to understand the basic process of this pathway, which is outlined in Figure 1. LDL‐C particles comprise an apoB_100_ molecule, which envelopes a core of cholesteryl esters and triacylglycerols, together with smaller amounts of other lipid species. During normal lipid regulation, these particles bind to LDL receptors expressed on the liver surface via their apoB molecule. The binding of LDL‐C to its receptor induces a rapid internalisation of the LDL‐C particle‐receptor complex into the endosome compartment of the cell, where the lipoprotein is broken down into its constituent lipids and amino acids. The LDL‐receptor then is either recycled back to the plasma membrane, or diverted to a lysosome and degraded, so that the LDL‐receptor is no longer available for recycling. Defects in any of these processes can therefore potentially cause FH.

The *LDLR* gene was the first gene found where mutations cause FH. It spans 45 kilobases (kb) on the short arm of chromosome 19 and comprises 18 exons that are transcribed and translated into five distinct domains, which form the cell surface LDL‐receptor (Hobbs et al. 1992). Any defect in the *LDLR* gene results in the loss of function of LDL‐receptors resulting in reduced LDL‐C uptake from blood and thus causes FH. FH‐causing mutations in the *LDLR* gene are found along the entire length of the gene. There are more than 2300 different variants identified in the *LDLR* gene, with a majority of them being exonic substitutions and small (< 100 bp) or large rearrangements (> 100 bp; Hobbs et al. 1992; Iacocca et al. 2018). Some variants are relatively common in specific countries such as Finland (Vuorio et al. 1997), South Africa (Leitersdorf et al. 1989) and in French Canadians (Hobbs et al. 1987), due to ‘founder effects’ because of past immigration and population expansion.

In the United Kingdom, and in many populations worldwide, gross deletions/insertions explain ∼10% of the molecularly defined FH index cases (Tosi et al. 2007; Futema et al. 2013). While deletions and insertions have been reported in all parts of *LDLR*, the majority are located in introns 1–8 and 12 through the 3’UTR, which corresponds to the distribution of ‘Alu’ repeat sequences in the gene (Leigh et al. 2008) and suggests these rearrangements are due to mis‐pairing and cross over at meiosis.

**FIGURE 2:**
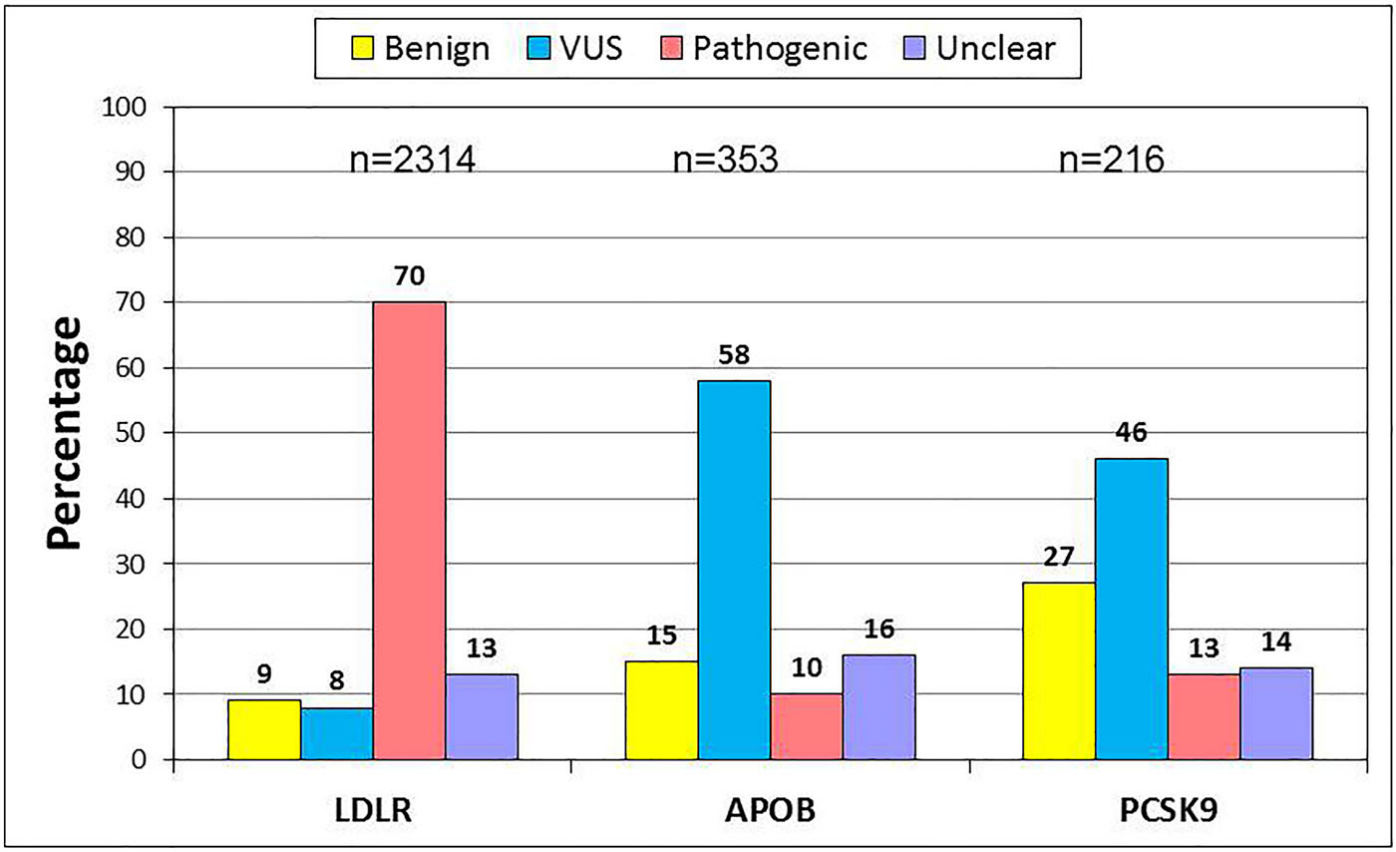
Published FH‐causing variants in *LDLR/APOB/PCSK9* coded using ACMG criteria (data from Iacocca et al. 2018). Variants are coded as benign/likely benign/variants of uncertain significance (VUSs)/likely pathogenic/pathogenic. Only pathogenic/likely pathogenic variants can be reported as FH‐causing, and all VUS need further work to determine whether they are FH causing or not.

It is clearly of importance to be able to assess the probability of whether a variant identified in a clinical setting or as an incidental finding in genomics projects is pathogenic or not. Predicting this is not always straightforward, especially for synonymous and missense variants. For *LDLR*, definitive proof that a variant is pathogenic requires either in vitro molecular assays or family studies. In vitro studies are necessary to examine the impact on transcription or correct splicing or LDL‐R expression, and although such studies have been reported for some variants, for the majority of *LDLR* variants, such data are lacking. In 2018, ClinVar published an update of all reported FH‐causing variants (Iacocca et al. 2018), using strict guidelines (Richards et al. 2015) to use the available evidence to designate a variant as benign or likely benign, a variant of uncertain significance (VUS) or likely pathogenic or pathogenic. The variant classification guidelines have been specifically modified for *LDLR* (Chora et al. 2022), and similar modifications for *APOB* and *PCSK9* are being developed. As shown in Figure 2, the analysis identified 2314 published *LDLR* variants of which 1620 (70%) were predicted to be pathogenic, while only 8% of reported *LDLR* variants were VUS using available evidence. The gold standard for proof of a variant being pathogenic requires family studies to see if other relatives who have inherited this variant have also shown high LDL‐C levels, while the relatives without the inherited variant have normal levels of LDL‐C. Such studies are time‐consuming and resource‐costly and are not widely carried out for FH.

One question of interest is what is the frequency of de novo mutations occurring in *LDLR* (or any of the other FH genes). Because of the high number of Alu sequences in the *LDLR* gene, it may be predicted that some of the deletions/insertions identified in individuals with clinical FH may be de novo, but we are unaware of any such reports. Only one paper has been published with definitive evidence of a de novo mutation (Pisciotta et al. 2002), where a subject was identified with the clinical phenotype of HeFH including early CHD, whose parents had normal plasma lipid values. The patient was heterozygous for a G > C transversion in exon 4, which results in the likely pathogenic variant p.(Cys109Ser). This variant was not carried by either parent, and non‐paternity was excluded using short tandem repeat polymorphic markers. Haplotype analysis indicated that this de novo mutation occurred in the paternal germ line. One likely reason for this paucity of evidence of de novo mutation is that, in order for a hypercholesterolaemic individual to be given the diagnosis of clinical FH and be referred for genetic testing, there has to be a family history of hypercholesterolaemia and/or early CHD, and for the carrier of a de novo mutation, this will be absent. It will be interesting to see if, in the future, more *LDLR* de novo mutations are detected by an increase of genetic screening in family trios as a result of greater availability of commercial tests or from incidental findings in health‐related research programmes.

### 2.2 APOB

Apolipoprotein B (apoB) is the major apolipoprotein of many lipoprotein particles, including LDL‐C, and it both supports the structure of the particle and functions as a ligand of the LDL‐receptor. The gene is located on chromosome 2p and spans more than 43 kb, comprising 29 exons, which encode a protein of 4563 amino acids (Austin et al. 2004). While truncation mutations in the *APOB* gene cause hypobetalipoproteinemia, mutations causing hypercholesterolemia are due to missense mutations that result in ligand‐defective apoB protein. Defective binding of LDL‐C from an individual with clinical FH, but no *LDLR* pathogenic variant, was reported in 1987 (Vega and Grundy 1986) and followed shortly by the identification of the genetic defect (Innerarity et al. 1987).

When an individual has a pathogenic variant in the *APOB* gene, the disorder is called familial defective ApoB‐100 (OMIM 107730) or FDB. The clinical and lipid phenotype of FDB patients overlap with the phenotype of FH in those carrying an *LDLR* mutation, but on average, they have a milder presentation than FH caused by *LDLR* mutations (Myant 1993; Myant et al. 1991). The most common mutation causing FDB alters the arginine at position 3527 to glutamine (p.(Arg3527Gln)) with the LDL containing ApoB‐Gln showing a very low affinity for the LDL‐receptor in in vitro assays and reduced clearance from the blood in turnover studies. The frequency of this mutation in (non‐Finnish) European populations reported in the gnomAD v4.1.0 database (https://gnomad.broadinstitute.org/) is 0.00049. This means that this variant is the single most common cause of the FH phenotype in subjects of European origin. Haplotype analysis using eight polymorphisms (including three microsatellites) at the *APOB* locus was used to determine the age of this variant. The data suggest that all European ancestry individuals who carry the 3537Gln variant carry this variant on a single haplotype and are descended from a common ancestor, with the distribution of the mutation being consistent with an origin in Western Europe 6000–7000 years (∼270 generations) ago. Following this, the mutation has spread northwards into Britain and Scandinavia, westwards at least as far as the Pyrenees, and eastwards towards Russia and the Balkans, being essentially absent from countries such as Finland and Greece (Myant et al. 1997).

A second mutation at this same codon has also been reported, p.(Arg3527Trp) (Gaffney et al. 1995), in a subject of Pakistani origin. The mutation co‐segregated with hyperlipidaemia in the family, and the LDL‐Trp showed poor binding to the LDL receptor. Data from gnomAD v4.1.0 show that this variant is mostly found in subjects of South and East Asian origin, where the frequency in the combined group is 0.00042. This means that this variant is likely the single most common cause of the FH phenotype in subjects of South and East Asian origin.

While a number of other pathogenic variants in *APOB* causing the clinical phenotype of FH have been reported, as shown in Figure 2, in the 2018 report, 353 variants in *APOB* had been published, of which 35 (10%) were designated as pathogenic with the majority of published *APOB* variants designated benign, likely benign or VUS (Iacocca et al. 2018). The large size and hydrophobic properties of apoB‐100 make it difficult to determine the effect of single amino acid changes; however, a recent study employing cryo‐electron microscopy has shed some new light on the interaction between LDL‐R and apoB_100_, expanding the binding sites between the two proteins, which might help to interpret the effect of DNA changes located within the regions (Reimund et al. 2024).

### 2.3 PCSK9

The *PCSK9* gene (proprotein convertase subtilisin/kexin type 9) encodes an enzyme that is involved in regulating the degradation of the LDL‐receptor protein in the lysosome of the cell, preventing it from being recycled to the cell surface. The gene is found on chromosome 1p and comprises 12 exons, covering 39 kb (17). The PCSK9 molecule is synthesised as an inactive proprotein and undergoes cleavage in the endoplasmic reticulum to produce an enzyme with the prodomain noncovalently bound to the catalytic site, preventing further enzyme action. PCSK9 is secreted mostly from the liver and its binding to the LDL receptor directs the receptor to the lysosome for degradation (Lagace et al. 2006).

The *PCSK9* gene was first identified as an FH gene by linkage analysis in a large French pedigree with individuals with clinical FH and no pathogenic variants in *LDLR* or *APOB* (Abifadel et al. 2003). This family contributed an LOD score of 4.26, and sequencing in this and other similar families identified different variants that co‐segregated with hypercholesterolaemia. Variants in the *PCSK9* gene that cause FH are gain‐of‐function variants that increase LDL receptor degradation and consequently reduce the number of receptors on the cell surface. More than 20 such variants have been reported worldwide (Abifadel et al. 2009), but the only common *PCSK9* variant in the United Kingdom is p.(Asp374Tyr), which accounts for 2% of all monogenic causes of FH. This variant is associated with a raised cholesterol level and a high risk of developing premature CHD, compared with a mutation in the *LDLR* gene (Humphries et al. 2006).

By contrast, loss‐of‐function mutations that inactivate the PCSK9 protein lead to less degradation of the LDL receptor and lower LDL‐C (Cohen et al. 2006). The most common of these variants, p.Arg46Leu, enhances the clearance of LDL‐C from the plasma and lowers cholesterol level in plasma. In European populations, approximately 3% of individuals are carriers of this variant, and because of their life‐long lower LDL‐C levels, they have ∼28% lower CHD risk (Benn et al. 2010).

Interestingly, a patient with the FH phenotype and a poor/limited response resistance to statin LLT has been found to have an entire duplication of the wild‐type *PCSK9* gene (Iacocca et al. 2018) which will clearly result in the FH phenotype because of higher plasma levels of the PCSK9 protein and greater hepatic LDL‐receptor degradation.

For *PCSK9*, predicting the functional consequences of an identified variant is more complex than for *LDLR* or *APOB*, since in silico prediction algorithms may predict that a missense change is likely to affect function, but cannot distinguish between a gain‐of‐function, possibly FH‐causing variant, and a loss‐of‐function, low LDL‐C variant. Figure 2 shows that using the ACMG guidelines, of 216 reported variants in *PCSK9*, only 28 were designated pathogenic while 46% of *PCSK9* variants were designated as VUS (Iacocca et al. 2018).

### 2.4 APOE

Apolipoprotein E (ApoE) is a 34 kDa liver‐derived multifunctional protein found associated with triglyceride‐rich chylomicrons and very LDLs (VLDL) and their remnants (Blum 1982; Mahley 1988). The gene for ApoE (*APOE*) is on chromosome 19 (but distant from the *LDLR* locus) and consists of 4 exons spanning 3.6 kb. ApoE mediates the high‐affinity binding of lipoproteins to the LDL‐R, which results in their clearance from circulation. It is a polymorphic protein with three major circulating isoforms E2, E3 and E4 (Utermann 1975) and lipid metabolism is isoform‐dependent. Isoforms are determined by a combination of two common non‐synonymous SNPs in exon 4 of the *APOE* gene (Rall et al. 1982). While allele frequencies differ across different ancestry groups, in all groups, the most common isoform is E3, with a cysteine residue at 112 and an arginine residue at 158 and is present in ∼79% of the European ancestry population. E4, the next most commonly encountered isoform (∼14% in European ancestry populations), has an arginine residue substituting cysteine at 112, and finally E2, which is present at a frequency of ∼7% in European ancestry populations, has a cysteine substituting arginine at residue 158. The resultant six common genotypes, in order of observed frequencies are ε3/ε3, ε3/ε4, ε3/ε2, ε2/ε4, ε4/ε4 and ε2/ε2.

While these *APOE* genotypes have a well‐documented influence on an individual's plasma lipid profile, they do not cause FH. However several recent studies have reported that one specific mutation p.(Leu167del) in *APOE* causes autosomal dominant FH (Marduel et al. 2013). The group analysed a large family with an autosomal dominant pattern of hypercholesterolaemia (and with no mutation in any of the usual FH genes) and used genome‐wide mapping, analysis of regional/functional candidate genes and whole exome sequencing (WES). This identified a 3‐base pair deletion (c.500_502delTCC), which resulted in the in‐frame deletion of the Leucine at residue 167. In silico analysis predicted the deletion would destabilise an alpha‐helix in the LDL‐R binding domain, which would likely lead to the decreased apoE level observed in the LDL particles of carriers and result in the decreased catabolism of LDLC from the blood. These findings have been confirmed and extended (Cenarro et al. 2016) by in vivo experiments showing the ApoE p.(Leu167del) causes down‐regulation of LDL‐receptors on hepatocytes, which would also result in slower clearance of apoE‐containing lipoproteins and lead to the FH phenotype.

We have recently reported (Bird et al. 2024) that the minor allele frequency (MAF) of this variant in the non‐FH subjects in the 100,000 genome (general population) sample was 8 × 10^−5^ (12 out of 77,275 individuals), with a highly significant enrichment of this variant in a sample of those individuals with clinical FH (10 out of 467 individuals, MAF = 0.01). Based on this work, we estimate that ∼2% of individuals in the United Kingdom with a clinical diagnosis of FH have this *APOE* variant as their monogenic cause.

### 2.5 The Contribution of the *LPA* Gene to the FH Phenotype

The lipoprotein(a) [Lp(a)] consists of an LDL‐C particle and a large glycoprotein, apolipoprotein(a) [apo(a)], which is covalently linked to apoprotein B‐100 in the LDL particle by a cysteine‐mediated single disulphate bond. Apo(a) has structural similarities to plasminogen, (a protein involved in coagulation and thrombolysis) and is encoded by the *LPA* gene on chromosome 6q26. Blood concentrations of Lp(a) are determined by their production rate from the liver, unlike LDL‐C, where it is the rate of removal by the hepatic LDL‐receptor pathway that determines concentration. Lp(a) concentration in blood is not influenced by diet or lifestyle and is largely determined by genotype at the *LPA* locus (Thompson and Seed 2014). While common variants in the *LPA* coding regions and in those that control gene expression and splicing are known to play a role, variations in the number of copies of the kringle IV‐2 repeat have the largest impact. The concentration of Lp(a) in the blood correlates inversely with the length of the protein, which is determined by the number of kringle IV‐2 repeats at the allele (i.e., the more repeats, the lower the concentration). Because of this, the final concentration of Lp(a) an individual has is the sum of the contribution of their maternal and paternal alleles. It has been estimated that the kringle IV‐2 repeat can explain about 61%–69% of the variability observed in Lp(a) levels in European populations (Boerwinkle et al. 1992). An Lpa‐genetic risk score (GRS) has been developed, using 42 SNPs at the locus, that is strongly associated with Lp(a) concentrations (Trinder et al. 2021).

An increased concentration of Lp(a) is a well‐established independent risk factor for CHD (Nordestgaard and Langsted 2016), and in Mendelian Randomisation studies, SNPs in the *LPA* region are strongly associated with CHD risk (Clarke et al. 2009). The risk of CHD is greatly increased if both LDL‐C and Lp(a) are elevated (Berry et al. 2023), and several studies have reported that individuals with a clinical diagnosis of FH have higher median Lp(a) concentrations than population‐matched healthy subjects (Olmastroni et al. 2023; Hedegaard et al. 2024).

We have recently published evidence that variation at the *LPA* locus is a significant contributor to the FH phenotype (Bird et al. 2024). Using whole genome sequencing (WGS) data generated by the 100,000 Genomes Project (100KGP), we analysed 536 FH patients diagnosed using the FH Simon Broome criteria, of whom 17.4% carried an FH‐causing variant. A genome‐wide association study (GWAS) was then performed between 443 FH variant‐negative unrelated FH cases and 77,275 control participants of the 100KGP using high coverage WGS data, which identified in the FH variant‐negative participants a single genome‐wide significant point at the *LPA* gene locus. Following this, GRSs for LDL‐C (LDL‐GRS) and lipoprotein(a) (Lpa‐GRS) were computed. As expected, FH variant‐negative participants had significantly higher LDL‐GRS but also a significantly higher Lpa‐GRS in comparison to the controls (*p* < 1.0 × 10^−16^ and *p* < 4.09 × 10^−6^). Similar associations were found in the monogenic FH with both the LDL‐GRS and Lpa‐GRSs being significantly higher than in controls. A high LDL‐GRS was observed in 36.4% of FH variant‐negative cases, with a high Lpa‐GRS in 18.5%, and with 7.0% having both high LDL‐GRS and high Lpa‐GRS.

This genome‐wide analysis of monogenic and polygenic FH causes confirms the previously reported role of the *LPA* locus as an additional gene where variation contributes to the FH phenotype (Berry et al. 2023; Trinder, DeCastro, et al. 2020). Taken together, these data suggest that individuals with a clinical diagnosis of FH (i.e., high LDL‐C and a family history of early CHD), but where the cause of their phenotype is due to variation at *LPA*, should not be given the clinical diagnosis of FH but rather be given the diagnosis of having a high‐Lp(a) disorder. The clinical relevance of this is that Lp(a) measurements should be included in the precision diagnosis of FH since this will identify individuals who will benefit most from specific therapies to lower Lp(a), which are in development (Milosavljevic et al. 2023).

### 2.6 *STAP1*: A Red Herring Gene for FH

In 2014, the gene *STAP1* was suggested to be a novel FH gene by linkage analysis in a large family from Holland (Fouchier et al. 2014) where many individuals had the clinical phenotype of FH, and no variants in any of the usual FH genes could be found. Three regions of the genome showed a logarithm of odds scores of 3.0 (θ = 0.0), and following the sequencing of gene exons in each region, the *STAP1* gene on chromosome 4p15.1‐q13.3 was identified as a potential new FH gene*. STAP1* codes for the signal transducing adaptor family member 1 and a potential functional variant was identified in the family and in other individuals with hypercholesterolaemia. The function of STAP1, also known as BRDG1 (BCR downstream signaling protein 1) or stem cell adaptor protein1, is largely unknown. STAP1 contains a Pleckstrin homology domain, a Src homology 2 domain and several tyrosine phosphorylation sites (Masuhara et al. 2000). However, none of these domains have any obvious involvement in lipid metabolism or hepatic LDL‐C clearance. Although these findings appeared robust, functional analysis (Loaiza et al. 2020) and failure in co‐segregation analysis (Lamiquiz‐Moneo et al. 2020) have definitively ruled out that variants in *STAP1* cause FH, demonstrating the degree to which caution must be used and a high threshold set for such candidates.

## 3 Prevalence of Monogenic Variants Causing FH in Different Ancestry Groups

**FIGURE 3:**
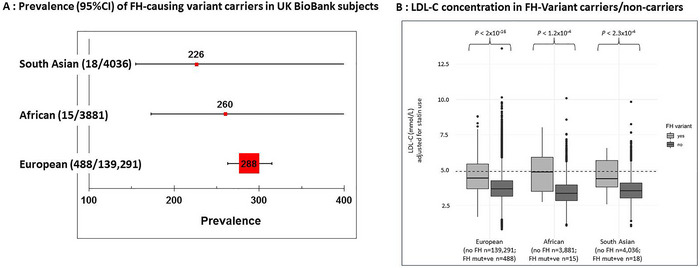
(A) Prevalence of FH‐causing variants and (B) LDL‐C concentrations in FH and non‐FH subjects in different ancestry groups from BioBank (data from Gratton et al. 2023). (A) The size of the data point is roughly proportional to the sample size. The lines indicate the 95% confidence intervals (CIs) of the point estimate. Since the CIs overlap, there is no statistically significant difference in the prevalence of FH‐causing variants between these three BioBank ancestry groups. (B) Histograms showing the mean (± the standard error) concentrations of LDL‐C in BioBank individuals of different ancestry groups carrying or not carrying an FH‐causing variant.

Anecdotally, it is thought that in the United Kingdom, compared to the prevalence of individuals of South Asian or African ancestry, there are significantly lower proportions of individuals with FH being managed in lipid clinics, suggesting that the prevalence of FH‐causing variants in these ancestry groups may be less than that reported in White subjects. We examined this in the UK BioBank sample (Gratton et al. 2023). FH‐causing variants in *LDLR, APOB* and *PCSK9* were determined using standard methods and genetic ancestry determined using principal component analysis. This resulted in 140,439 White British, 3906 African and 4067 South Asian individuals with WES data and a full lipid profile. Median LDL‐C concentrations were respectively 3.68, 3.36 and 3.54 mmol/L (overall difference *p* < 2.2 × 10^−16^). As shown in Figure 3A, there was no significant difference in the prevalence of an FH‐causing variant, being 1/288, 1/260 and 1/226 (overall *p*‐value = 0.57), suggesting that the lower prevalence of these ancestry groups in UK lipid clinics is due to under‐diagnosis. As expected, in each ancestry group, carriers of an FH‐causing variant had significantly higher LDL‐C concentration than non‐carriers (Figure 3B), showing that the under‐diagnosis is not primarily due to a lack of penetrance of the variants. These findings have an important message for strategies to ensure equality of access of FH management and reduction of CHD risk to all ancestry groups.

## 4 The Polygenic Cause of the FH Phenotype

While in those with the highest clinical suspicion of FH (Simon Broome Definite FH or a DLCN score > 8) between 40% and 80% have a monogenic cause, in those with a lower clinical suspicion the detection rate is usually 20%–30% (Taylor et al. 2010). In the patients with clinical FH but where no FH‐causing variant has been found, a polygenic aetiology should be considered due to the co‐inheritance of a greater‐than‐average number of common LDL‐C raising genetic variants (SNPs). In 2010, a meta‐analysis of GWAS data identified 95 loci involved in determining lipid levels (Teslovich et al. 2010), and we have used a 12‐SNP LDL‐C GRS including the weighted sum of the LDL‐C‐raising alleles, where weights are the effect sizes from GWAS. Data from UK patients (Talmud et al. 2013) and in several international collaborations (Futema et al. 2015; Mariano et al. 2020) suggest that in more than 80% of those with a clinical diagnosis of FH but with no detectable monogenic cause, the polygenic predisposition is most likely driving the hypercholesterolaemia phenotype. Interestingly, in those with a confirmed mutation, the score frequency distribution was intermediate between the healthy subjects and the clinical FH mutation‐negative group, suggesting that even in patients with an identified mutation, a polygenic lipid‐raising profile is added to their clinical phenotype. The GRS also impacts CHD risk in those with FH, and individuals who are heterozygous for an FH‐causing variant but also have a high LDL‐GRS are at higher risk of CHD than those with a variant but with a low GRS (Trinder, Paquette, et al. 2020).

Knowing an individual's LDL‐C risk score has clinical utility. We have shown that, compared to those heterozygous for an FH‐causing variant, the degree of atherosclerosis in the carotid artery and of calcification in the coronary arteries is significantly lower in those with a high LDL‐GRS even though LDL‐C concentration is similar in the two groups (Sharifi et al. 2017). Using a 223 SNP LDL‐GRS in the BioBank subjects, it has been shown that the LDL‐GRS is strongly associated with the risk of CHD as expected because of its association with LDL‐C concentration. However, while individuals in the highest decile of the score have the highest concentration of LDL‐C and the highest risk of CHD, their risk is considerably less than those who are heterozygous for an FH‐causing variant (Trinder, Francis, et al. 2020). The likely explanation for this higher risk is that those heterozygous for an FH‐variant have been exposed to high LDL‐C from birth and thus have a higher accumulated LDL‐C‐Burden than those with no such variant (Vuorio et al. 2013). Thus, while the polygenic hypercholesterolaemia group are at modest risk of CHD and require LLT, the higher CHD risk in those with monogenic FH requires the intensive LLT recommended by FH guidelines (Mach et al. 2020).

## 5 Possible Novel Monogenic Causes Suggested by GWAS and WES or WGS Approaches

Can we expect that WES or WGS will identify additional FH‐causing variants in the no‐variant clinical FH individuals? The current data suggest that any ‘new‐FH gene’ is likely to explain a smaller proportion of cases than either the *APOE* or *PCSK9* loci (i.e., less than ∼1% of clinical FH individuals) since if such a common cause of FH were present it would have been already identified by the studies carried out to date. In this case, very large studies will be required to have adequate statistical power. At the extreme, it may be that every family now being investigated will have a different genetic cause for the FH phenotype. This will make proof of causality very dependent on family co‐segregation and demonstrating the functional impact of every detected variant as the only way to confirm the involvement of the locus. Clearly at the present time, we lack the high‐throughput assays of LDLR function and LDL‐C particle affinity needed to address this in a cost‐effective manner, though such assays are being developed (Graça et al. 2023; Islam et al. 2023; Jasiecki et al. 2023). One possibility that has yet to be fully explored is that variation in understudied regions of the loci for the current FH genes may be making a significant additional contribution. For example, variation in deep intronic regions may contribute (Reeskamp et al. 2018) or in up‐stream or down‐stream regions of the gene, which are potentially involved in controlling gene expression. A large‐scale WGS association analysis with blood lipids suggested that *LDLR* introns 2, 3, 16 and 17 harbour rare variants that demonstrated to have a significant (FH‐like) impact on LDL‐C (Selvaraj et al. 2022). Although the existing next‐generation sequencing technologies can identify variants in non‐coding parts of FH genes, the biggest challenges include variant annotation and interpretation. One possibility is to collate the information from databases such as ENCODE, which provides tissue‐specific data on the open chromatin regions, where variants might alter the binding of transcription factors (ENCODE Project Consortium 2004). In addition, examining the available WGS data from no‐variant clinical FH individuals, after the exclusion of all individuals with a high enough LDL‐GRS, might identify novel regions of the FH‐causing genes with pathogenic variants or possibly suggest novel FH‐causing loci.

As an example of the current data, WES was reported on 213 individuals from 41 pedigrees with likely Mendelian inheritance of very high or very low concentrations of LDL‐C. In nine families, likely pathogenic variants in known FH‐causing genes (*APOB, APOE, LDLR, LIPA* and *PCSK9*) were found, with no monogenic cause identified in the remainder and a high LDL‐GRS being present in many families (Stitziel et al. 2015).

Our previous attempt employing WES of variant‐negative FH patients highlighted a few potential novel causes (Futema et al. 2014). Exomes of 125 unrelated DFH patients were sequenced as part of the UK10K project. Of these, 23 carried an *LDLR* pathogenic variant, two carried the *APOB* pathogenic variant and 29 had a high LDL‐GRS. Patients with these explained causes of FH were excluded from further analysis. Initial analysis of the remaining 71 samples focussed on genes associated with LDL‐C concentrations in GWAS meta‐analysis, but no statistically significant signals were identified. After this, a gene‐based burden test for an excess of rare (frequency < 0.005) or novel variants in these 71 cases versus 1926 controls was performed. Again, no major novel locus for FH was detected, with no gene having a likely functional variant in more than three patients, However, an excess of novel variants was found in 18 genes, of which the strongest candidates were *CH25H* and *INSIG2*, which both code for proteins known to have a direct role on the transcription of the *LDLR* gene and thus the expression of LDL‐receptors in liver cells. In both genes, several potentially functional variants were identified in individuals with clinical FH, but unfortunately no co‐segregation studies were possible to confirm or refute them as novel FH‐causing genes.

While most of the loci identified in this study have not been confirmed in subsequent studies, one of the loci identified was the *RBM25* gene. *RBM25* encodes the RNA binding motif protein 25, which is involved in the regulation of alternative mRNA splicing, via the spliceosome. This protein is part of the U2‐splicosome complex and has recently been shown to be involved in the posttranscriptional regulation of the LDL receptor (Zanoni et al. 2022). By using small inhibitory RNA methods to systematically knock down gene expression in cultured hepatocytes, a number of important proteins involved in controlling *LDLR* expression were identified. Of these, 15 were involved in the function of the U2‐spliceosome, identifying this complex and its cognate genes as potential novel FH‐causing genes. In support of this, SNPs of the *RBM25* gene show a significant association with LDL‐cholesterol in several different population studies, and three rare variants were identified in individuals with FH. Expression of these variants in a liver cell line demonstrated between 15%–28% lower expression of the LDL receptor on the cell surface, supporting their potential role in causing FH in these individuals.

Using Bayesian genetic testing for rare variants in whole genomes, in a sample of 469 FH patients, the *RAB35* gene was significantly associated with FH, with a potentially functional variant also co‐segregating with hypercholesterolaemia in one affected relative (Greene et al. 2023). This gene codes for the Ras‐related protein Rab‐35, which encodes a GTPase which participates in the traffic of recycling endosomes towards the plasma membrane, suggesting an additional pathway where variants may cause clinical FH.

## 6 Conclusion

Although it was initially described as a single‐gene monogenic disorder, we now know that the genetic architecture of the FH phenotype is considerably more complex and genetically heterogeneous. As well as loss‐of‐function and gain‐of‐function pathogenic variants in four genes involved in the hepatic clearance of LDL‐C (*LDLR/APOB/PCSK9/APOE*) common variants in these same genes, as well as in at least 150 other gene loci spread throughout the genome (Vanhoye et al. 2023), can combine to elevate LDL‐C concentrations to those seen in monogenic FH. While individuals with this ‘polygenic hypercholesterolaemia’ do have an elevated CHD risk, this is lower than in those with monogenic FH, and risk can be lowered more successfully by LLT. The FH phenotype can also be mimicked by having a combination of SNPs at the *LPA* locus, which results in high concentrations of the LDL‐C‐related particle Lp(a). Individuals with this disorder will benefit from a differential diagnosis since the usual LLTs appropriate for FH are ineffective in lowering Lp(a). Finally, while there are some credible reports of additional novel monogenic causes of FH, all of these are making only a very small contribution to the overall FH patient numbers and none have been fully confirmed by replication and co‐segregation studies.

**FIGURE 4:**
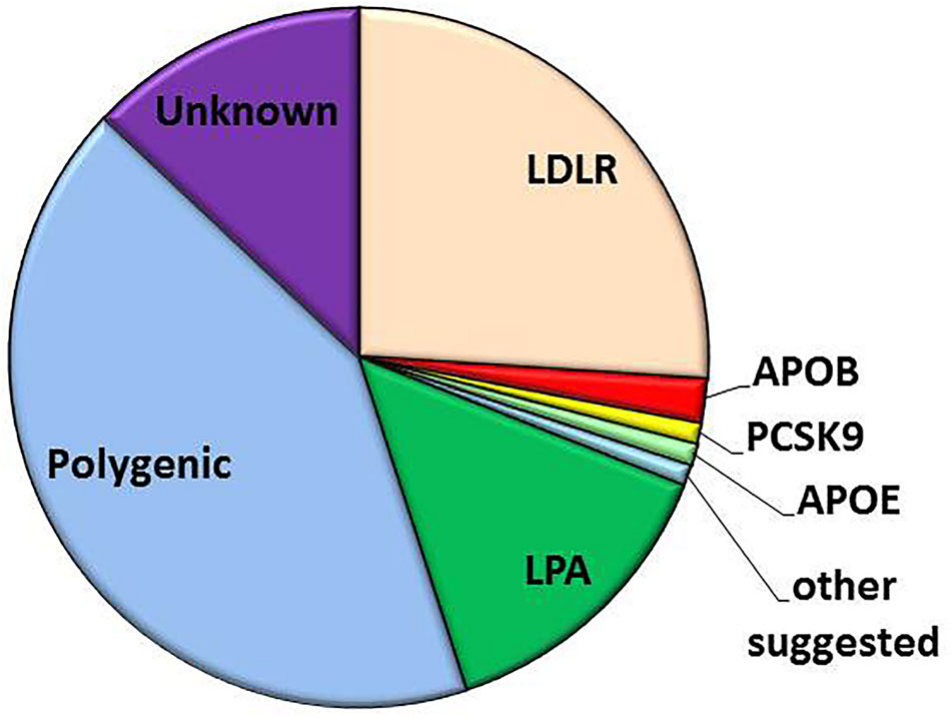
Pie chart with the estimated relative contribution of monogenic, polygenic and unknown causes to the FH phenotype. The relative sizes of the slices have been estimated assuming (1) 30% of subjects with clinical FH have a monogenic cause in *LDLR/APOB/PCSK9/APOE*, with the relative proportions within this 30% being 88%, 6%, 3% and 3%. Of the remaining 70%, we estimate 60% (i.e., 42% of the total) have an LDL‐GRS in the top 6 deciles and have polygenic hypercholesterolaemia, while (3) 20% of the remainder (i.e., 14%) have a high LPA‐GRS and have a high Lp(a) concentration mimicking the FH phenotype. We suggest that an additional 1% at most are likely to have a monogenic FH‐cause in one of the novel genes so far identified (e.g., *INSIG2/CH25H/RMB25/RAB35*) leaving ∼13% with an unexplained cause.

In Figure 4, we attempt, in cartoon form, to summarise the likely relative contribution of both currently known monogenic and polygenic contributions to the FH phenotype, as well as the likely proportion of the novel as yet unconfirmed (and likely rare) monogenic gene causes. As discussed previously, while the proportion of those with clinical FH who are found to carry an FH‐causing variant will vary depending on the ratio of Definite FH versus Possible and Probable FH, based on UK data (Humphries et al. 2023), we have assumed that not more than 30% will have a genetic confirmation of monogenic FH. It seems unlikely that more than an additional 1% at most will have a monogenic FH‐cause in one of the novel genes so far identified. Of the remaining 70%, we estimate 60% (i.e., 42% of the total) have an LDL‐GRS in the top 6 deciles and have polygenic hypercholesterolaemia (Talmud et al. 2013), while 20% of the remainder (i.e., 14%) have a high LPA‐GRS and have a high Lp(a) concentration mimicking the FH phenotype (Hedegaard et al. 2024). This suggests that there is a small proportion (10%–15%) of individuals with the FH phenotype that have a yet unidentified cause, though to what extent this is environmental or genetic is unclear. However, to carry out research to examine this will require very large‐scale sequencing studies of clinical FH‐variant‐negative/low LDL‐GRS and LPA‐GRS individuals, as well as family studies and in vitro functional assays to identify and confirm potential novel signals.

Despite this improved understanding of the genetic architecture of the FH phenotype, and the clear data demonstrating the prevalence of individuals carrying an FH‐causing variant of ∼1/300, worldwide, the vast majority of individuals with FH remain undiagnosed (Nordestgaard et al. 2013). In the future, WGS (Humphries et al. 2023) or universal screening for elevated cholesterol concentrations in newborns, in infancy or childhood, with conformation by genetic testing, will identify new probands (Ramaswami et al. 2024). With appropriate counselling of families, these can be used as the starting point for relative testing to find those who also have genetic FH. However, such approaches raise financial, logistic and ethical issues that are beyond the scope of this review.

## Author Contributions

Steve Eric Humphries produced the first draft, and Marta Futema commented and amended this and referenced the final manuscript.

## Conflicts of Interest

Steve Eric Humphries is the medical director of a UCL Spin‐off company (StoreGene) that offers genetic testing for cardiovascular risk including for FH. Steve Eric Humphries and Marta Futema also report payment for expert testimony from Verve Therapeutics.

## Data Availability

Data sharing is not applicable to this article as no new data were created or analyzed in this study.
